# Assessment of incompleteness of Mortality Information System records on deaths from external causes in the state of Rio Grande do Sul, Brazil, 2000-2019

**DOI:** 10.1590/S2237-96222023000200006

**Published:** 2023-07-17

**Authors:** Juliane de Souza Barbosa, Luiza Tartaro, Lucas da Rosa Vasconcelos, Marcela Nedel, Jéssica Ferri Serafini, Suely Garcia Suslik Svirski, Leandra Soares de Souza, Marilyn Agranonik

**Affiliations:** 1 Universidade do Vale do Rio dos Sinos, Programa de Pós-Graduação em Saúde Coletiva, São Leopoldo, RS, Brazil; 2 Universidade do Vale do Rio dos Sinos, Faculdade de Medicina, São Leopoldo, RS, Brazil; 3 Secretaria de Planejamento, Governança e Gestão do Estado do Rio Grande do Sul, Departamento de Economia e Estatística, Porto Alegre, RS, Brazil

**Keywords:** Information Systems, Death Certificate, Vital Statistics, External Causes, Ecological Study, Sistemas de Información, Certificado de Defunción, Estadísticas Vitales, Causas Externas, Estudio Ecológico, Sistemas de Informação, Atestado de óbito, Estatísticas vitais, Causas externas, Estudo ecológico

## Abstract

**Objective:**

to evaluate the incompleteness of Mortality Information System (*Sistema de Informações sobre Mortalidade* - SIM) data on deaths from external causes (ECs) in the state of Rio Grande do Sul, Brazil, 2000-2019.

**Methods:**

This was an ecological study, using SIM data on all deaths from external causes and, specifically, from transport accident, homicides, suicides and falls; the analysis of the trend of incompleteness was performed by means of Prais-Winsten regression, with a 5% significance level.

**Results:**

A total of 146,882 deaths were evaluated; sex (0.1%), place of death (0.1%) and age (0.4%) showed the lowest incompleteness in 2019; the proportion of incompleteness showed a decreasing trend for the place of death and schooling, an increasing trend for marital status and a stable trend for age and race/skin color, among all types of death evaluated.

**Conclusion:**

the variables analyzed reached a high degree of completion; with the exception of marital status and schooling, for which unsatisfactory scores persisted for deaths from ECs, both total and by subgroups.

## Resumo

**Objetivo:**

avaliar a incompletude dos dados do Sistema de Informações sobre Mortalidade (SIM) para óbitos por causas externas (CEs) no Rio Grande do Sul, Brasil, 2000-2019.

**Métodos:**

estudo ecológico, com dados do SIM para a totalidade das CEs e, especificamente, por acidentes de transporte, homicídios, suicídios e quedas; analisou-se a tendência da incompletude via regressão de Prais-Winsten, com nível de significância de 5%.

**Resultados:**

146.882 óbitos foram avaliados; sexo (0,1%), local de ocorrência do óbito (0,1%) e idade (0,4%) mostraram as menores incompletudes, em 2019; a proporção de incompletude apresentou tendência decrescente para local de ocorrência do óbito e escolaridade, crescente para estado civil e estável para idade e raça/cor da pele, entre todos os tipos de óbito avaliados.

**Conclusão:**

as variáveis analisadas alcançaram alto grau de preenchimento; à exceção do estado civil e da escolaridade, para as quais persistiram escores insatisfatórios para óbitos por CEs, totais e por subgrupos.

## Resumen

**Objetivo:**

evaluar la incompletitud de los datos del Sistema de Información de Mortalidad (SIM) para las defunciones por causas externas (CEs) en Rio Grande do Sul, Brasil, 2000-2019.

**Métodos:**

estudio ecológico con datos del SIM por causas externas, totales y por accidentes de tránsito, homicidios, suicidios y caídas; se utilizó la regresión de Prais-Winsten para evaluar la tendencia de incompletitud, con un nivel de significancia del 5%.

**Resultados:**

se evaluaron 146.882 muertes; sexo (0,1%), lugar de muerte (0,1%) y edad (0,4%) mostraron menos incompletitud en 2019; la tendencia de la proporción de incompletitud disminuyó para el lugar de ocurrencia y la educación, aumentó para el estado civil y se mantuvo estable para edad y raza/color del piel para todo tipo de muertes evaluadas.

**Conclusión:**

las variables analizadas lograron alta calidad de información, con excepción del estado civil y educación, que persistieron con incompletitud insatisfactoria para las muertes por CE (total y subgrupos).

**Table:** 

Study contributions	
**Main results**	Sex, age, place of death, and race/skin color showed excellent levels of incompleteness for deaths from external causes (ECs) - below 5% for the entire period - while education showed 35.2%, and marital status, 11.3%, in 2019.
**Implications for services**	High level of incompleteness may modify the profile of deaths from ECs, causing biases for studies with data from the Mortality Information System (*Sistema de Informações sobre Mortalidade* - SIM). It is necessary to qualify the completion of some fields of the Death Certificate.
**Perspectives**	The monitoring of the completion of the SIM data, with continuous qualification of the information, will provide excellent levels of incompleteness and characterization of the most vulnerable groups, in a reliable way, for all variables.

## INTRODUCTION

External causes (ECs) have occupied the top positions among the leading causes of death in recent years in Brazil. Among the Brazilian states, Rio Grande do Sul ranked seventh in deaths from ECs in 2019.^1^

The analysis of epidemiological and sociodemographic characteristics of deaths from ECs helps the planning and implementation of specific actions, according to social and health conditions.^2^ ^,^ ^3^ In this context, the Mortality Information System (*Sistema de Informações sobre Mortalidade* - SIM) becomes an essential tool, both in monitoring the number of deaths and in identifying the most vulnerable groups, providing contributions to public policy design.^4^ ^,^ ^5^ In this sense, it is necessary to achieve a high degree of completeness of the variables in this information system. The degree of incompleteness is an indicator that measures the non-completion of records. Taking into consideration, especially, the inequalities observed in Brazil, whether among Brazilian states or macro-regions,^6^ or among the causes of death,^7^ ^-^ ^9^ the degree of incompleteness is the indicator par excellence for continuous monitoring of non-completion of records of sociodemographic variables included in the Death Certificate (DC).

Among the deaths from ECs, the state of Rio Grande do Sul stands out for being one of the ten states with the highest number of deaths attributed to these causes and for having showed the highest suicide rate in the country since 2008.^1^ Therefore, the analysis of the epidemiological profile of these deaths by means of the SIM points to the need to know the degree of completeness of the variables in this information system.

The objective of this study was to evaluate the incompleteness of SIM data on deaths from ECs recorded in the state of Rio Grande do Sul, Brazil, between 2000 and 2019.

## METHODS

An ecological time series study was conducted using the SIM records on all deaths from ECs - chapter XX of the International Statistical Classification of Diseases and Related Health Problems (ICD-10) - and, specifically, the subgroups of deaths due to transport accidents (chapters V01-V99 of ICD-10), homicides (X85-Y09), suicides (X60-X84) and falls (W01-W20), which occurred in the state of Rio Grande do Sul between 2000 and 2019. The four causes of death specified in this study, were chosen because they are the most prevalent among the ECs in the state, accounting for 85% of these deaths in 2019.^1^

The state of Rio Grande do Sul is located in the Southernmost part of Brazil, its capital is Porto Alegre and its geographical area is 281,707.1 km², where 497 municipalities are distributed. It had an estimated population of 11,466,630 inhabitants in 2021.^10^ In 2019, in Rio Grande do Sul, the Family Health Strategy coverage was 59.0%, and Primary Health Care coverage was 74.3%.^11^ The information used in this study was obtained from the SIM, via the Brazilian National Health System Information Technology Department (*Departamento de Informática do Sistema Único de Saúde* - DATASUS) website, in May 2021.^1^ SIM has a coverage of approximately 100% of the deaths registered in the Southern region of the country.^12^

The degree of incompleteness was defined as the percentage of information that was not filled in, considering the data ignored (code “9” in the SIM manual) or left blank. The degree of incompleteness was defined according to the cut-off points proposed by Romero and Cunha:^13^ excellent (incompleteness below 5%); good (5% to 9.9% incompleteness); regular (10% to 19.9%); poor (20% to 49.9%); and very poor (50% incompleteness or more). The incompleteness of the variables included in the DC regarding the following sociodemographic characteristics was evaluated: sex (male; female; ignored); age (in full years: under 1; 1 to 4; 5 to 9; 10 to 14; 15 to 19; 20 to 29; 30 to 39; 40 to 49; 50 to 59; 60 to 69; 70 to 79; 80 years and older; ignored); race/skin color (White; mixed race; Black; Asian; Indigenous; ignored); schooling (in completed years of study: 0; 1 to 3; 4 to 7; 8 to 11; 12 or more; ignored); marital status (single; married; widowed; judicially separated; other; ignored); and place of death (hospital; other health facility; home; public road; others; ignored).

Initially, the percentage change (Δ%) in the proportion of incompleteness between the beginning and the end of the period selected for the study was calculated by dividing the difference in the percentage of incompleteness between 2019 and 2000 by the percentage of incompleteness in 2000. Subsequently, aiming to assess the trend of incompleteness in the time series for each variable, Prais-Winsten regression, corrected for first-order autocorrelation, was used.^14^ This analysis allowed us to interpret whether the trend was increasing, decreasing, or stationary. The dependent variable was the proportion of incompleteness; and the independent variable was the years of the period studied. The significance level was 5%.

Statistical analysis was performed using the Stata software, version 14.0. This was an ecological study, based on aggregate secondary data, in the public domain, aimed at protecting the identity of the participants and, therefore, it was exempted from the approval of a Research Ethics Committee (REC). It is noteworthy that all other recommendations included in the National Health Council (*Conselho Nacional de Saúde* - CNS) Resolution No. 466, of December 12, 2012, were followed.

## RESULTS

Between 2000 and 2019, there were 146,882 deaths from ECs in the state of Rio Grande do Sul, representing 9.5% of the deaths that occurred during the period. Sex, age, place of death and race/skin color showed an excellent degree of incompleteness (< 5%) for all causes of death evaluated (Table 1). In 2019, the lowest incompleteness observed in the completion of sociodemographic characteristics were related to sex, place of death and age, among deaths due to suicide and falls. The degree of incompleteness of marital status ranged from good (2000-2010) to regular (2011-2019) for all ECs, and specifically, transport accidents, homicides and suicides, reaching 6.6% in 2019. Regarding deaths due to falls, filling in of marital status ranged from excellent (2000-2006) to good (2007-2019). Schooling presented the worst performance in the completion, initially with a very poor classification (2000-2010) and finally poor (2011-2019): in 2019, the last year of the study period, the incompleteness of schooling remained high, ranging from 26.0% for deaths from falls to 38.8% for deaths from transport accidents.

**Table 1:** -Percentage of incompleteness of selected variables from the Mortality Information System on deaths from external causes, the state of Rio Grande do Sul, Brazil, 2000-2019

Sociodemographic variables and cause s of death	Incompleteness (%)																			
	2000	2001	2002	2003	2004	2005	2006	2007	2008	2009	2010	2011	2012	2013	2014	2015	2016	2017	2018	2019
	6,485	6,522	6,853	6,808	6,932	6,869	6,909	7,136	7,344	7,289	7,209	7,079	7,537	7,693	7,876	7,842	8,379	8,586	7,973	7,561
Age																				
External causes	0.3	0.4	0.3	0.3	0.3	0.2	0.4	0.6	1.1	0.7	0.6	0.8	0.6	0.5	0.3	0.5	0.3	0.4	0.6	0.5
Transport accidents	0.2	0.3	0.3	0.2	0.3	0.1	0.2	0.3	0.5	0.2	0.1	0.3	0.4	0.2	0.1	0.1	0.1	0.1	0.2	0.1
Homicides	0.3	0.3	0.4	0.2	0.2	0.2	0.5	0.9	1.4	1.3	1.2	1.4	0.9	1.1	0.4	0.8	0.6	0.9	1.1	0.9
Suicides	0.3	0.1	-	0.2	0.1	0.1	0.1	0.1	-	-	-	0.2	-	0.1	-	0.1	-	0.1	0.1	-
Falls	0.4	-	-	-	-	-	-	-	-	0.4	-	-	-	-	-	-	-	-	-	-
Place of death																				
External causes	1.2	1.0	0.8	0.5	0.6	0.7	0.8	0.4	0.5	0.6	0.4	0.4	0.4	0.3	0.3	0.2	0.2	0.3	0.2	0.1
Transport accidents	0.6	0.5	0.3	0.2	0.1	0.1	0.1	0.1	-	0.3	0.1	0.2	0.5	0.2	0.2	-	-	0.2	0.1	-
Homicides	1.5	1.3	1.2	0.8	1.0	1.2	0.9	0.4	0.9	0.5	0.5	0.4	0.3	0.5	0.3	0.2	0.3	0.5	0.2	0.2
Suicides	1.5	1.0	0.5	0.3	0.6	0.6	0.8	0.7	0.5	0.9	0.7	0.3	0.2	0.4	0.5	0.4	0.3	0.2	0.2	-
Falls	0.4	0.7	-	0.3	-	-	0.8	-	0.4	-	0.2	0.3	-	-	-	-	-	0.2	0.1	0.0
Race/skin color																				
External causes	2.0	1.5	1.2	0.9	0.9	0.8	1.0	1.1	1.4	1.1	1.2	1.6	1.4	1.7	1.4	1.7	1.7	1.4	1.8	1.9
Transport accidents	1.5	1.3	0.6	0.5	0.7	0.2	0.6	0.6	1.3	0.9	1.1	1.4	0.9	1.2	1.0	1.6	0.8	1.1	0.9	1.1
Homicides	1.9	2.1	1.3	0.6	0.8	0.6	0.8	1.1	1.6	1.0	1.3	1.2	2.1	2.1	1.5	1.4	1.7	1.0	1.5	2.1
Suicides	1.9	1.2	0.4	0.5	0.1	0.3	0.7	1.0	0.9	0.8	0.4	1.1	1.0	0.6	1.1	0.8	0.9	0.7	0.9	1.0
Falls	4.9	0.3	2.3	1.8	1.1	1.2	0.5	0.4	1.2	1.6	1.0	2.7	1.2	3.1	1.3	3.6	2.4	3.3	4.0	2.7
Marital status																				
External causes	6.6	6.4	7.0	7.0	6.4	7.1	7.7	9.0	8.8	8.5	8.9	11.4	11.3	12.4	10.8	11.1	9.9	10.1	12.5	11.3
Transport accidents	6.1	5.4	6.3	5.8	5.2	6.1	7.3	7.8	7.8	7.2	8.4	11.6	11.9	13.2	11.8	11.1	10.1	11.3	12.3	10.0
Homicides	7.0	6.1	7.2	5.9	4.5	6.4	7.1	6.9	7.6	7.5	7.8	9.4	9.6	10.0	9.3	9.5	8.1	8.4	11.9	11.8
Suicides	4.4	3.2	4.3	5.1	4.6	5.4	5.6	5.9	6.1	7.0	7.5	12.1	11.2	12.7	9.5	11.3	9.0	8.8	10.6	9.6
Falls	3.8	2.4	3.4	3.9	4.1	2.9	4.0	5.9	5.3	3.8	7.3	6.8	7.8	9.5	7.4	8.3	7.9	8.8	10.3	8.4
Schooling																				
External causes	56.2	56.2	58.2	57.7	56.0	57.9	56.3	57.6	55.2	54.1	52.8	44.4	39.4	38.2	38.1	40.2	36.4	36.4	37.7	35.2
Transport accidents	58.5	56.9	59.4	58.1	56.1	55.6	55.9	55.6	52.9	51.8	51.3	46.0	44.5	42.7	40.5	43.1	40.1	39.8	39.9	38.8
Homicides	60.0	58.2	62.2	61.8	60.0	63.7	59.8	63.0	60.2	57.9	56.8	42.9	34.8	35.6	34.6	38.5	33.5	34.1	38.1	34.8
Suicides	54.3	54.8	55.0	55.9	55.1	55.5	52.3	54.7	51.3	49.2	50.1	44.2	40.8	40.5	40.4	42.6	39.1	38.2	37.7	35.7
Falls	55.1	56.0	56.9	55.4	49.7	52.8	48.7	49.3	49.6	49.4	50.6	38.4	40.9	34.5	35.3	31.8	30.2	29.1	30.7	26.0

Note: The dash ( - ) represents numeric data equal to 0.0 (zero) not resulting from rounding.

It could be seen different trends regarding incompleteness rates in the completion of sociodemographic characteristics, according to causes of death (Table 2). Sex and age showed a stable trend for the proportion of incompleteness among deaths from transport accidents and deaths from homicides. With regard to sex, as well as age, it was not possible to analyze the trend in deaths due to suicide and deaths from falls due to the large number of years at zero level of incompleteness. The proportions of incompleteness for place of death and schooling showed a decreasing trend in all causes of death analyzed (p-value < 0.05). Filling in of race/skin color remained stable for ECs throughout the period, among the subgroups of causes of death. On the other hand, there was an increasing trend of incompleteness of marital status for all causes of death evaluated (p-value < 0.001).

**Table 2:** - Percentage change and temporal trend of the proportion of incompleteness of sociodemographic variables from the Mortality Information System on deaths from external causes (N = 20), the state of Rio Grande do Sul, Brazil, 2000-2019

Sociodemographic variables and cause s of death	Δ%^a^	95%CI^b^	Adjusted R²^c^	p-value^d^	Trend
Age					
External causes	46.1	45.9;46.3		0.451	Stable
Transport accidents	-71.7	-71.9;-71.5	0.149	0.097	Stable
Homicides	192.4	191.9;192.8	0.031	0.169	Stable
**Place of death**					
External causes	-92.2	-92.4;-91.9	0.796	< 0.001	Decreasing
Transport accidents	-82.1	-82.5;-81.7	0.294	0.044	Decreasing
Homicides	-84.6	-85.2;-84.0	0.780	< 0.001	Decreasing
Suicides	-89.0	-89.7;-88.2	0.527	0.002	Decreasing
Falls	-78.4	-79.2;-77.7	0.297	0.010	Decreasing
**Race/skin color**					
External causes	-4.2	-4.7;-3.8	0.398	0.306	Stable
Transport accidents	-25.8	-26.5;-25.0	0.119	0.430	Stable
Homicides	8.3	7.4;9.2	0.182	0.465	Stable
Suicides	-47.1	-48.0;-46.1	0.181	0.792	Stable
Falls	-45.7	-48.5;-42.9	0.123	0.074	Stable
**Marital status**					
External causes	70.1	69.2;71.1	0.591	< 0.001	Increasing
Transport accidents	64.4	62.6;66.2	0.309	0.005	Increasing
Homicides	67.2	65.4;69.0	0.569	< 0.001	Increasing
Suicides	118.6	116.6;120.6	0.375	0.001	Increasing
Falls	120.6	117.9;123.4	0.871	< 0.001	Increasing
**Schooling**					
External causes	-37.4	-39.0;-35.8	0.863	< 0.001	Decreasing
Transport accidents	-33.7	-37.0;-30.5	0.949	< 0.001	Decreasing
Homicides	-42.1	-45.2;-39.0	0.744	0.002	Decreasing
Suicides	-34.3	-38.2;-30.4	0.915	< 0.001	Decreasing
Falls	-52.8	-59.3;-46.3	0.899	< 0.001	Decreasing

a) Δ%: Percentage change in the proportion of incompleteness between 2000 and 2019; b) 95%CI: 95% confidence interval for Δ; c) Adjusted R²: Adjusted coefficient of determination; d) P-value for the F-test of the Prais-Winsten regression model.

## DISCUSSION

This study found discrepancies in the degree of incompleteness among the variables analyzed, with some fields showing excellent completion, while others still had a higher degree of incompleteness. Among the causes of death investigated, there was no difference in the level of completion for the same variable. There was a decrease in incompleteness for place of death and schooling, and an increase in marital status. The other variables showed stable incompleteness throughout the period.

Sex, age, race/skin color and place of death showed excellent completion for all causes of death evaluated. These results strongly suggest that these variables can be used in demographic and epidemiological analyses of deaths from ECs in the state of Rio Grande do Sul. Other studies, conducted in the Southeast region of the country in 2007,^7^ ^,^ ^8^ and in the state of Bahia in 2010,^9^ found excellent levels of completion only for sex and age in deaths from breast cancer and suicide. On the other hand, the completion of place of death differs among cities, states or macro-regions of the country: in the Southeast region, in 2007,^8^ and in the Northeast region, in 2010, and in the states of Bahia^9^ and Pernambuco,^15^ the filling in was excellent, while in Fortaleza, the capital of the state of Ceará, it was very poor.^16^

The decreasing trend of incompleteness for race/skin color was also found in other Brazilian regions, with a reduction of at least 50% in incompleteness between 2000 and 2015.^6^ However, studies that analyzed the incompleteness of this variable according to specific causes of death found percentages higher than those observed in this study. In Fortaleza, one of the worst incompleteness was found for race/skin color, 38% in 2010,^16^ while Romero et al. identified states such as Alagoas, Bahia, Minas Gerais and Espírito Santo with scores below the category excellent, in 2015.^6^ Therefore, despite the lower percentages identified in the state of Rio Grande do Sul for the completion of race/skin color, regional inequalities persist in the completion of the DC. 

However, marital status and schooling did not obtain good or excellent scores. As for marital status, there was an increase in the degree of incompleteness in the period. Similar results were observed in the Southeast region in 2006,^8^ and in the state of Pernambuco in 2010.^15^ Throughout the period analyzed, schooling showed the highest scores, remaining with approximately 35% of incompleteness in 2019. The state of Rio Grande do Sul showed better results in this study, when compared to those observed in Fortaleza in 2010,^16^ although the proportion of incompleteness in the state of Rio Grande do Sul is still below that found by Rios et al. in the state of Bahia and by Melo and Valongueiro in the state of Pernambuco, also in 2010.^9^ ^,^ ^15^ It is worth highlighting the importance of schooling and marital status, sociodemographic variables pointed out as indicators of social vulnerability.^3^ ^,^ ^17^ Studies that use variables with low completeness may incur biases, distorting the profile of deaths, which may be reflected in the absence of public policies aimed at the actual needs of the population.

In view of large variations in the completion of different variables, it is noteworthy that initially, the Ministry of Health classified the fields of DC as indispensable, essential or secondary, adding a greater degree of importance to the variables “sex”, “type of death”, “underlying cause” and “municipality of occurrence”.^18^ However, since 2001, the institution has recommended the completion of all the variables included in the DC.^19^ Despite this change, the initial classification of these fields may have caused a mistaken view among the professionals responsible for filling them out, that some variables are less important than others. The way health professionals take into consideration the importance of the variables included in the DC may impact on differences in data completeness among regions, states and municipalities. A study conducted in the state of Rio Grande do Norte, in 2013, when comparing the knowledge of physicians, professors, residents and medical students about the DC, observed similar performance among professors and students, and their knowledge was lower than that of the group of residents.^20^ This result indicates unsatisfactory knowledge, with low rates of completion of certain variables, possibly explained by the lack of training, during academic life, on the importance of completing the DC.^21^

This study analyzed only one Brazilian state and it was limited to one of the dimensions of data quality, incompleteness, focusing on deaths from external causes. There are also limitations inherent to the use of secondary data, such as the possibility of underreporting of deaths, duplicity and reliability of DC information. It is noteworthy that the SIM coverage in the states of the South region is approximately 100%,^12^ and continuous actions implemented by Rio Grande do Sul Health Department have allowed a reduction close to zero, for duplicate data, as well as they have sought to increase their reliability. The results of this research contribute to greater visibility of one of the aspects of the quality of information on mortality statistics in the state of Rio Grande do Sul, based on a long historical series on deaths. The analysis of incompleteness over a 20-year period allowed a reliable observation of its trend.

The research showed a decreasing or stable trend in incompleteness of most of the SIM variables related to deaths that occurred in the state of Rio Grande do Sul during the study period. However, large differences remain in the degree of incompleteness of these variables. The improvement of the quality of the data entered into the SIM depends on the structure of the services involved, the management tools and the perception of the professionals regarding the completeness of these records. In conclusion, the authors of this note recommend the conduction of continuous qualification courses and sensitization of health professionals and medical students in the state of Rio Grande do Sul on the importance of filling in all fields of the DC.
